# Hybridisation of perovskite nanocrystals with organic molecules for highly efficient liquid scintillators

**DOI:** 10.1038/s41377-020-00391-8

**Published:** 2020-09-07

**Authors:** Sangeun Cho, Sungwoo Kim, Jongmin Kim, Yongcheol Jo, Ilhwan Ryu, Seongsu Hong, Jae-Joon Lee, SeungNam Cha, Eun Bi Nam, Sang Uck Lee, Sam Kyu Noh, Hyungsang Kim, Jungwon Kwak, Hyunsik Im

**Affiliations:** 1 Division of Physics and Semiconductor Science, Dongguk University, Seoul, 04620 Republic of Korea; 2 Department of Radiation Oncology, Asan Medical Center, Seoul, 05505 Republic of Korea; 3 Department of Energy and Materials Engineering, Dongguk University, Seoul, 04620 Republic of Korea; 4 Department of Physics, Sungkyunkwan University, Suwon, 2066 Republic of Korea; 5 Department of Bionano Technology and Department of Applied Chemistry, Hanyang University, Ansan, 15588 Republic of Korea

**Keywords:** X-rays, Quantum dots

## Abstract

Compared with solid scintillators, liquid scintillators have limited capability in dosimetry and radiography due to their relatively low light yields. Here, we report a new generation of highly efficient and low-cost liquid scintillators constructed by surface hybridisation of colloidal metal halide perovskite CsPbA_3_ (A: Cl, Br, I) nanocrystals (NCs) with organic molecules (2,5-diphenyloxazole). The hybrid liquid scintillators, compared to state-of-the-art CsI and Gd_2_O_2_S, demonstrate markedly highly competitive radioluminescence quantum yields under X-ray irradiation typically employed in diagnosis and treatment. Experimental and theoretical analyses suggest that the enhanced quantum yield is associated with X-ray photon-induced charge transfer from the organic molecules to the NCs. High-resolution X-ray imaging is demonstrated using a hybrid CsPbBr_3_ NC-based liquid scintillator. The novel X-ray scintillation mechanism in our hybrid scintillators could be extended to enhance the quantum yield of various types of scintillators, enabling low-dose radiation detection in various fields, including fundamental science and imaging.

## Optics: Hybrid liquid scintillator with enhanced light output

A hybrid liquid scintillator could be used in low-dose radiation detectors for use in imaging applications and scientific research. Highly sensitive X-ray detection is increasingly being used in industrial and military applications and for fundamental scientific research. Although liquid scintillators are more resilient to damage from exposure to intense radiation than crystalline or plastic scintillators, they have relatively low density and low radioluminescence quantum yields, both of which are critical for achieving high resolution and contrast in X-ray imaging. Now, a team of Korean researchers led by Hyunsik Im from Dongguk University has created a liquid scintillator detector made from colloidal perovskite metal halide nanocrystals and organic molecules with a significantly enhanced quantum yield. The novel device could see use in a wide range of X-ray technologies that require high-performance detectors and imagers.

## Introduction

Highly sensitive X-ray detection is becoming increasingly important in areas from everyday life to industry, the military, and scientific research^1–4^. Scintillation materials convert X-ray^5^, γ-ray^6^, and particle radiation into visible or ultraviolet (UV) light. Among the various properties of scintillation materials, quantum yield (or light output) is the one most closely associated parameters with both the efficiency and resolution of detectors. Because the quantum yield depends on the nature of the incident particles and photons with varying degrees of energy, a proper scintillation material is chosen according to the type of application. Compared with crystalline or plastic scintillators, liquid scintillators generally have better resistance to damage arising from exposure to intense radiation while providing excellent area/volume scalability^7,8^; consequently, liquid scintillators are used for various purposes, such as in β-ray spectroscopy, radioactivity measurements, and particle physics^9,10^. However, despite the above advantages, liquid scintillators have relatively low density and low radioluminescence (RL) quantum yield, both of which are crucial in achieving high resolution and contrast in X-ray imaging. As a result, liquid scintillators have rarely been utilised in radiation imaging.

Recently, metal halide perovskite materials, including both bulk crystals of organic inorganic hybrid perovskites and nanocrystals (NCs), have been demonstrated^11–14^ to efficiently convert X-ray photons into charge carriers or visible photons^15–19^. In particular, fully inorganic perovskite NCs have advantages such as highly emissive X-ray-generated excitonic states^20^, ultrafast radiative emission rates^21^, and resistance against high-energy radiation^22^, all of which are essential for highly efficient and durable X-ray scintillators. Moreover, perovskite NCs have high optical sensitivity in response to exposure to X-rays and high X-ray absorption efficiency^22,23^. Perovskite NCs are also commonly uniformly dispersed in nonpolar liquid media for use in liquid scintillators. However, despite their unique properties being superior to those of commercially manufactured scintillators, for example, Tl-doped CsI^24^ and Gd_2_O_2_S^25^, perovskite NCs still require further improvements in their quantum yield for practical applications. Here, we report an experimental investigation of highly efficient X-ray scintillation and significantly enhanced quantum yields of liquid scintillators consisting of perovskite metal halide CsPbA_3_ (A: Cl, Br, I) NCs and C_15_H_11_NO (2,5-diphenyloxazole: PPO) organic molecules in soft and hard X-ray regimes and demonstrate their use in high-resolution X-ray imaging. We propose a new type of mechanism for substantially enhancing the scintillation quantum yield, which is accomplished by hybridising different scintillation nanomaterials.

## Results

### Hybrid CsPbA_3_ liquid scintillators and radiography

**Fig. 1:**
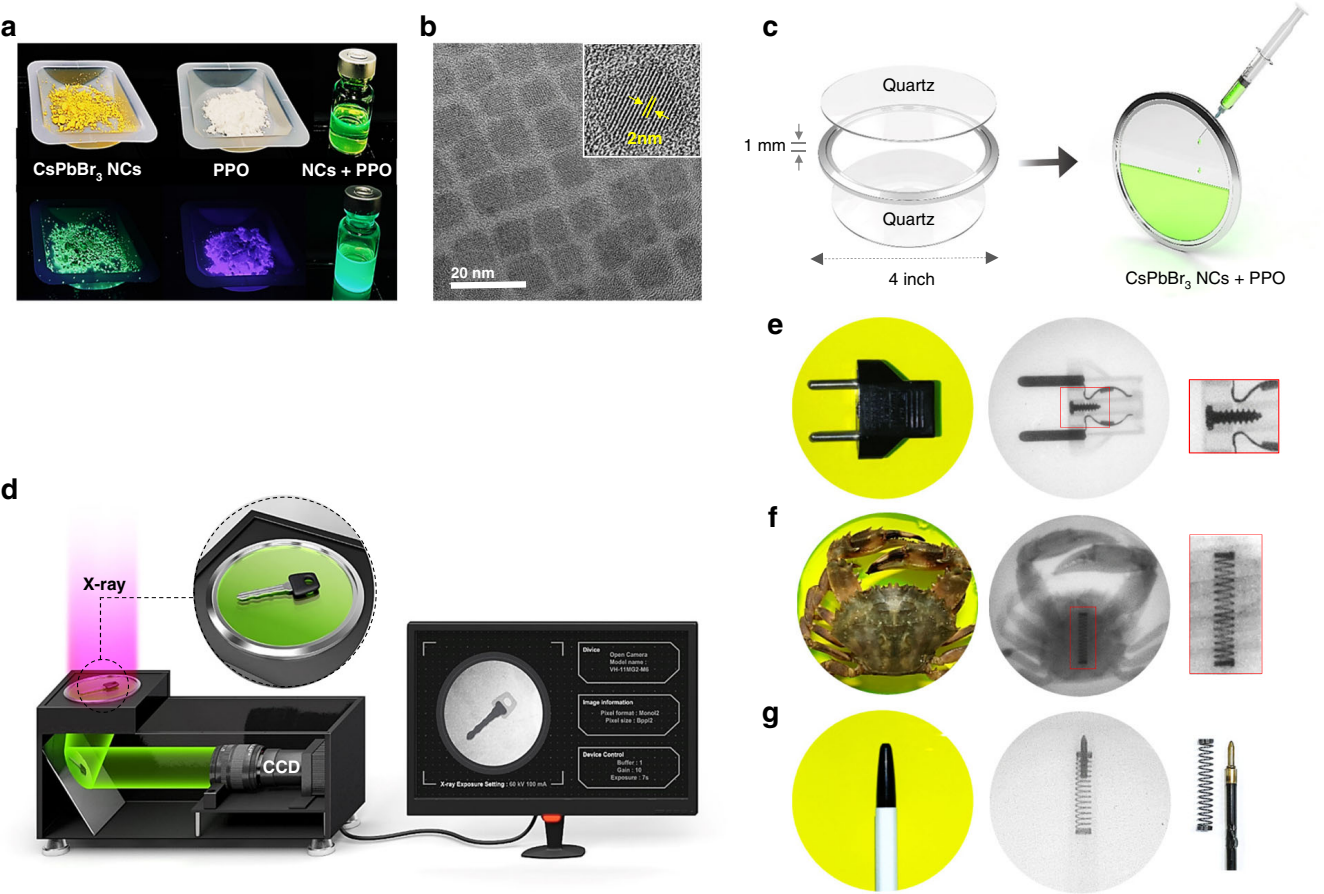
X-ray radiography using colloidal CsPbBr3 nanocrystals (NCs) hybridised with 2,5-diphenyloxazole (PPO). **a** Photographs of CsPbBr_3_ NCs, PPO and colloidal hybrid CsPbBr_3_ NCs+PPO in octane under white light (upper column) and UV illumination (lower column). **b** TEM image of the CsPbBr_3_ NCs. The inset shows a high-resolution TEM image of a single CsPbBr_3_ NC. The size distribution of the CsPbBr_3_ NCs is shown in Supplementary Fig. S1. **c** X-ray flat panel detector consisting of the hybrid CsPbBr_3_ NCs+PPO scintillator dispersed in octane and sandwiched by two quartz windows. The thickness of the colloidal hybrid scintillator is 1mm. **d** Schematic of the real-time X-ray imaging system consisting of a charge-coupled device (CCD) camera and a specially designed liquid film panel containing the colloidal hybrid CsPbBr_3_ NCs+PPO scintillator. **e**–**g** Optical and X-ray images of an electric power plug, a biological specimen (crab) containing a piece of metal, and a ball point pen containing the same piece of metal on the scintillator panel

The hybrid liquid scintillators were manufactured by dispersing CsPbA_3_ NCs and PPO in octane without precipitation (Fig. 1a). The perovskite NCs were synthesised via a hot injection method^26–28^ (see “Methods” for details). Transmission electron microscopy (TEM) measurements revealed that the as-synthesized NCs have a cubic shape with an average size of 12 nm (Fig. 1b). The optical and structural properties of the perovskite NCs were investigated using photoluminescence (PL), ultraviolet-visible (UV-Vis) spectroscopy, X-ray diffraction (XRD) measurements, and TEM images (Supplementary Figs. S1 and S2)^29–31^. To quickly evaluate the suitability of the CsPbBr_3_ NCs+PPO material as a scintillator for X-ray imaging, we imaged a wide range of biological and inorganic specimens with X-rays using a liquid scintillator panel (Fig. 1c) combined with a charge-coupled device (CCD) camera (Fig. 1d). For radiographic measurements, the specially designed display panel was used. The colloidal hybrid CsPbBr_3_ NCs+PPO solution was sandwiched by two quartz windows with 4-inch diameters. The X-ray images were taken at an accelerating voltage of 70 kVp. To demonstrate X-ray imaging, the concentrations of the CsPbBr_3_ NCs and PPO in octane were set at 25 mg/ml and 10 mg/ml, respectively. An object was placed on the panel detector, and an X-ray-excited optical image was projected through a mirror onto the CCD. As will be discussed in further detail, the CsPbBr_3_ NCs+PPO scintillator was selected to demonstrate the X-ray imaging because the CsPbBr_3_ NCs have excellent durability and the strongest RL intensity. As shown in Fig. 1e–g and Supplementary Fig. S3, the metal structures within the biological and plastic specimens were clearly imaged on the liquid scintillator panel.

### Enhanced radioluminescence in hybrid CsPbA_3_ scintillators

**Fig. 2:**
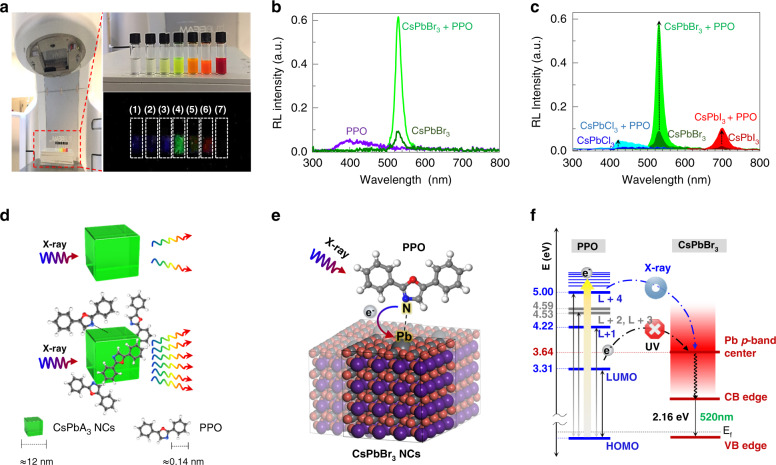
Enhanced RL of CsPbA3 NCs+PPO (A: Cl, Br, I) hybrid materials in octane. **a** X-ray generator used for X-ray imaging and RL measurements. The magnified photographs show the hybrid CsPbA_3_ NCs+PPO samples in ambient light and under X-ray irradiation. The material compositions of samples 1 through 7 are (1) CsPbCl_3_, (2) CsPbCl_2_Br, (3) CsPbCl_1_Br_2_, (4) CsPbBr_3_, (5) CsPbI_1_Br_2_, (6) CsPbI_2_Br_1_, and (7) CsPbI_3_. **b** RL spectra of the hybrid CsPbBr_3_ NCs+PPO, CsPbBr_3_ NCs, and PPO scintillators. **c** RL spectra of the hybrid CsPbA_3_ NCs+PPO and CsPbA_3_ NCs scintillators. **d** Schematic illustration of the RL of a CsPbA_3_ NC and a CsPbA_3_ NC hybridised with PPO. **e** Schematic diagram describing the hybridisation of a CsPbBr_3_ NC with PPO. The negatively charged N in the PPO binds to the positively charged Pb sites on the (001) surface of the CsPbBr_3_ NC. **f** DFT calculations of the energy level alignment for the proposed mechanism of enhanced RL in the hybrid CsPbBr_3_ NCs+PPO scintillator. Under X-ray irradiation, a high-energy electron (*e*^*-*^ in the solid circle) generated in the PPO moves to CsPbBr_3_ NCs

Figure 2a shows photographs of the X-ray imaging system and colloidal CsPbA_3_ NCs+PPO scintillators in the presence of white light and under X-ray irradiation (accelerating voltage: 6 MVp). During X-ray exposure, the CsPbBr_3_ NCs+PPO scintillator exhibited the brightest RL and emitted a green colour. As anticipated, the hybrid CsPbBr_3_ NCs+PPO scintillator exhibited the highest RL intensity in both the soft and hard X-ray regimes (Supplementary Fig. S4).

Figure 2b shows a comparison of the RL spectra emitted from the CsPbBr_3_ NCs (25 mg/ml), PPO (10 mg/ml), and hybrid CsPbBr_3_ NCs + PPO scintillators. The hybrid NCs+PPO scintillator exhibited strong RL that was several times stronger than those emitted by other scintillators. The hybrid NCs + PPO and pure NCs scintillators had the same RL peak positions, indicating that adding PPO does not significantly affect the emission energy of the CsPbBr_3_ NCs while enhancing their RL intensity. Another interesting observation is that the RL signal of PPO completely disappears in the spectrum of the hybrid NCs+PPO scintillator, suggesting the likelihood of X-ray-induced charge transfer from PPO to the CsPbBr_3_ NCs. We have experimentally demonstrated that the RL spectrum of a powder mixture containing the same amounts of PPO and CsPbBr_3_ NCs without octane exhibited two resolved RL emissions, each corresponding to PPO and CsPbBr_3_ NCs (Supplementary Fig. S5) and that the PPO peak is not suppressed in the PL spectrum of the hybrid NCs+PPO scintillator under UV irradiation (see Supplementary Fig. S6); collectively, these findings support the proposed mechanism that PPO plays a key role in enhancing the RL of the CsPbA_3_ NCs in octane. The surface hybridisation of halide perovskite NCs with PPO is highly feasible in a nonpolar liquid solvent medium such as octane. The same dramatic RL enhancement was also observed with the hybrid CsPbCl_3_ NCs + PPO and CsPbI_3_ NCs + PPO scintillators (Fig. 2c).

### Scintillation mechanism

In lead halide perovskite NCs, the photoelectric interaction between incident high-energy X-ray photons and heavy lattice atoms produces high-energy electrons, and these energetic electrons subsequently generate secondary high-energy carriers^32,33^. The hot carriers then undergo a thermalisation process, producing numerous low-energy excitons, and, consequently, high-energy X-ray photons are converted to visible low-energy photons via direct-bandgap luminescence^23^. For our hybrid CsPbA_3_ NCs+PPO scintillators, X-ray-induced energetic electrons generated from PPO can transfer to the CsPbA_3_ NCs via surface hybridisation and amplify the number of energetic electrons in the NCs, thereby enhancing the RL from the CsPbA_3_ NCs with a significantly improved quantum yield (Fig. 2d).

Density functional theory (DFT) calculations were performed to simulate the surface hybridisation of CsPbBr_3_ NCs with PPO and elucidate the origin of the improved quantum yield in the hybrid CsPbBr_3_ NCs+PPO scintillator in terms of X-ray-induced charge transfer from PPO to the NCs. For hybridisation of the CsPbBr_3_ NCs with PPO, the PPO must compete with the oleic acid (OA) ligand bound to the CsPbBr_3_ NC surfaces via surface reactions. Thus, we first compared the binding energies of PPO and OA on the CsPbBr_3_ NC surfaces and assessed how well the desorbed OA could be dissolved in octane.

Neutral PPO and anionic OA showed binding energies of −1.03 eV and −0.30 eV on the Pb site, respectively, and anionic OA had a larger solvation free energy of −36.05 kcal/mol compared with the value of −9.82 kcal/mol for PPO in octane solvent. The calculated results revealed that PPO, with its relatively large binding energy, can replace the OA on the CsPbBr_3_ NC surface and that the desorbed OA can be stabilised in octane with a large negative solvation free energy (Supplementary Table S1). Therefore, the formation of the hybrid CsPbBr_3_ NCs+PPO in octane was facilitated by the strong interaction between PPO and Pb ion sites through N-Pb bonding (Supplementary Figs. S7 and S8). XPS measurements of CsPbBr_3_ NCs, PPO, and CsPbBr_3_ NCs+PPO provide strong evidence for N-Pb bonding (Supplementary Fig. S9).

We analysed the energy level alignment between PPO and the CsPbBr_3_ NCs and the frontier orbital distributions. X-ray-induced charge transfer was allowed when the excited state of PPO was much higher than the conduction band state of the CsPbBr_3_ NCs. In particular, the large contribution of N and Pb in forming N-Pb bonds that led to the aligned states of PPO and the CsPbBr_3_ NCs effectively led to charge transfer from PPO to the CsPbBr_3_ NCs (Fig. 2e and Supplementary Fig. S10). Here, we used the *p*-band centre of the Pb atom as the representative conduction band state of the CsPbBr_3_ NCs because the valence 6*p*-orbital of Pb is involved in the N–Pb bond and is distributed over a wide range of conduction bands with various contributions. The energy level alignment in Fig. 2f revealed that the lowest unoccupied molecular orbital (LUMO) state of PPO and the *p*-band centre of the Pb atom were located at 3.31 eV and 3.64 eV, respectively. Energy levels are denoted based on the aligned Fermi energy (E_f_) of the hybrid CsPbBr_3_ NCs+PPO at 0 eV. In addition, the N atom in PPO significantly contributed to LUMO, LUMO+1, and LUMO+4; therefore, the LUMO+1 and LUMO+4 states, which were located above the *p*-band centre of the Pb atom and had large contributions from the N atom, could effectively induce charge transfer from PPO to the CsPbBr_3_ NCs. This implies that a sufficiently high-energy source, such as X-ray irradiation, is required to induce charge transfer from the excited states above the LUMO of PPO to the Pb *p*-orbital of the CsPbBr_3_ NCs.

Consequently, the characteristic structural and electronic features of the hybrid CsPbBr_3_ NCs+PPO scintillator resulted in selective charge transfer under X-ray irradiation, eventually enhancing the scintillation quantum yield. On the other hand, an excited electron in PPO cannot move to an NC upon UV illumination because the energy levels of the allowed states in the NC are too high. This is consistent with the experimental observation that low-energy UV light cannot enhance the quantum yield in the hybrid NCs+PPO scintillator (Supplementary Fig. S11).

### Characterisation of radioluminescence

We then measured the RL spectra of the CsPbA_3_ NC (25 mg/ml)+PPO (10 mg/ml) hybrid scintillators as a function of dose rate (Supplementary Fig. S12). The measured RL emission exhibited a linear response to the X-ray dose rate, which is a desirable feature of a good scintillator for X-ray imaging and dosimetry. We also measured the X-ray response characteristics of the hybrid scintillator upon excitation with a single X-ray photon from a portable coin-type ^60^Co source (Supplementary Fig. S13). The extracted fast scintillation decay time τ was 60–100 ns for the CsPbA_3_ NCs+PPO hybrid scintillators, which is much shorter than that for the bulk CsI:Tl (on the order of μs). The fast RL decay time of the hybrid scintillators is also expected to act as a favourable trait for use in medical radiography.

**Fig. 3:**
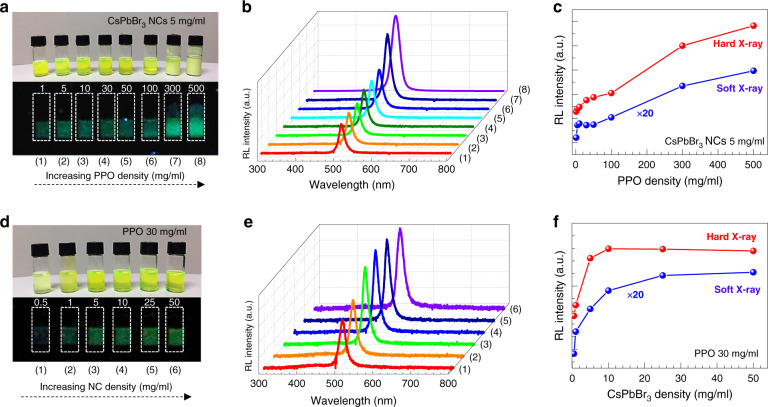
Radioluminescence of the hybrid CsPbBr3 NCs+PPO scintillators with different density ratios. **a** Photographs of the hybrid CsPbBr_3_ NCs+PPO scintillators in ambient light and under X-ray irradiation. The PPO density was increased from 1 to 500mg/ml. The PPO densities of samples 1 through 8 were (1) 1, (2) 5, (3) 10, (4) 30, (5) 50, (6) 100, (7) 300, and (8) 500mg/ml. **b** RL spectra of hybrid scintillator samples 1 through 8 in the hard X-ray regime. **c** RL peak intensity for the hybrid CsPbBr_3_ NCs+PPO scintillators as a function of PPO density. CsPbBr_3_ NC density: 5mg/ml. **d** Photographs of the hybrid CsPbBr_3_ NCs+PPO scintillators. The CsPbBr_3_ NC density was increased from 0.5 to 50mg/ml. The NC densities of samples 1 through 6 were (1) 0.5, (2) 1, (3) 5, (4) 10, (5) 25, and (6) 50 mg/ml. **e** RL spectra of hybrid scintillator samples 1 through 6 in the hard X-ray regime. **f** RL peak intensity of the hybrid CsPbBr_3_ NCs+PPO scintillator as a function of CsPbBr_3_ NC density. PPO density: 30 mg/ml

We further quantitatively investigated how PPO contributes to the RL of the CsPbBr_3_ NCs+PPO hybrid scintillator by varying the concentration ratio of the NCs and PPO. As shown in the photographs (Fig. 3a) under X-ray irradiation (accelerating voltage: 6 MV_p_), the RL emission from the hybrid NCs+PPO scintillator became brighter as the PPO density was increased for a fixed NC density of 5 mg/ml. We measured the RL spectra of the hybrid NCs+PPO scintillators in the soft (dose rate of 37.4 mGy s^−1^ at an accelerating voltage of 50 kVp) and hard X-ray regimes (Fig. 3b, Supplementary Fig. S14a), plotted the measured RL peak intensity as a function of the PPO density (Fig. 3c), and observed a linear relationship between the RL intensity and PPO density. The scintillation efficiency of a hybrid CsPbBr_3_+PPO scintillator was enhanced with increasing PPO density (Supplementary Fig. S15). Without CsPbBr_3_ NCs, the RL intensity of the pure PPO liquid scintillator decreased at high PPO densities (>10 mg/ml), which was likely due to scintillation quenching (namely, self-absorption)^34,35^ (Supplementary Fig. S16). When the PPO density was greater than 50 mg/ml in the colloidal hybrid scintillator, a yellowish-green dense precipitate was formed. As the PPO density was further increased to above the critical value of ~500 mg/ml, the hybrid NCs+PPO material in octane completely transformed into an opaque dense precipitate that emitted a very strong RL.

We also carried out similar measurements while increasing the CsPbBr_3_ NC density for a fixed PPO density of 30 mg/ml and observed that precipitates were not formed. In contrast, as the CsPbBr_3_ NC density increased, the RL emission quickly saturated in both the soft and hard X-ray regions, as shown in the photographs (Fig. 3d) and RL spectra (Fig. 3e and Supplementary Fig. S14b). The observed features are summarised in Fig. 3f, in which the measured RL peak intensity is plotted as a function of NC density.

### X-ray imaging performance

**Fig. 4:**
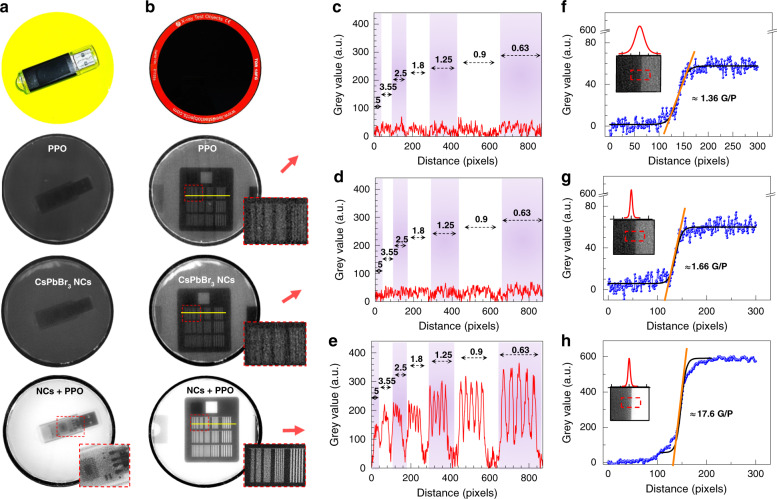
X-ray imaging with significantly enhanced resolution using the hybrid CsPbBr3 NCs+PPO scintillator. **a** Photograph of a data storage device on a homemade X-ray flat panel detector consisting of the hybrid CsPbBr_3_ NCs+PPO scintillator and X-ray images taken using the PPO, CsPbBr_3_ NCs and hybrid CsPbBr_3_ NCs+PPO scintillators. The densities of the PPO and CsPbBr_3_ NCs were 10 mg/ml and 25 mg/ml, respectively. **b** Photograph of the Leeds test objects on the homemade X-ray panel, and X-ray images taken using the PPO, CsPbBr_3_ NCs, and hybrid CsPbBr_3_ NCs+PPO scintillators. The X-ray images were taken at a voltage of 70kVp. **c**–**e** X-ray line pair profiles along the yellow line in Fig. 4b. The numbers (0.63−5) indicate lp/mm. **f**–**h** Edge spread function (ESF) along the lines in the X-ray images (as shown in the inset) taken using the PPO, CsPbBr_3_ NCs, and hybrid CsPbBr_3_ NCs+PPO scintillators from the top. G/P: grey value/pixel

To directly confirm any enhancement in the X-ray image quality when using the hybrid CsPbBr_3_ NCs+PPO liquid scintillator, we recorded the X-ray images of a portable data storage device using PPO, CsPbBr_3_ NC, and hybrid CsPbBr_3_ NCs+PPO scintillators (Fig. 4a). The hybrid NCs+PPO scintillator produced notably clearer X-ray images than those of PPO the CsPbBr_3_ NCs. The spatial resolution and image quality of the scintillation materials were quantitatively evaluated using a radiography test phantom^36,37^ (Leeds test objects, model: TOR 18FG, Supplementary Fig. S17). Figure 4b shows X-ray images of the test objects. The imaging performances of the scintillators were comparatively evaluated by counting the maximal number of resolvable line pairs per millimetre (lp/mm) and checking the abruptness of the contrast changes at the boundary. Figure 4c–e shows the intensity variation along the yellow lines in the X-ray images of the line patterns. The largest detectable lp/mm of the hybrid scintillator was at least 3.5 lp/mm, which was several times greater than those of the pure halide perovskite NCs and PPO scintillators.

Figure 4f–h show the edge spread functions (ESFs) at the boundary of the test phantom, indicated by the red dashed boxes in the insets, which characterise the sharpness of the images. The abrupt change in intensity is reflected by the slope across the boundary. The measured slope was 17.6 grey value/pixel for the hybrid NCs+PPO scintillator, which was much larger than those for the other materials (range, 1.36–1.66 grey value/pixel) (pixel size: 9 μm). The line spread functions (LSFs) extracted from the ESFs (red curves above the insets) also indicated how sharp the image was near the boundary in terms of the full width at half maximum (FWHM)^38^. The estimated FWHM of the NCs+PPO hybrid material was 7.6 pixels, which is much smaller than those of the other scintillators (range: 16–32 pixels). The image contrast is a measure of how clearly an object is distinguishable and can be assessed using the following expression:

1 $${\mathrm{Contrast}}\left( \% \right) = 100 \times \left( {I_{{\mathrm{Object}}} - I_{{\mathrm{Background}}}} \right)/\left( {I_{{\mathrm{Object}}} + I_{{\mathrm{Background}}}} \right)$$

where *I*_Object_ and *I*_Background_ represent the RL intensities of the object and adjacent material near the boundary, respectively. The contrast near the boundary was 33% for the hybrid NCs+PPO scintillator and 12−13% for the other scintillators.

Ageing and deterioration of the hybrid NCs+PPO scintillator were examined by repeating the X-ray imaging measurements in the same environment after a year (Supplementary Fig. S18) and after continuous irradiation with a very high-energy X-ray for a prolonged period (Supplementary Fig. S19). The colloidal hybrid scintillator exhibited almost no degradation in performance, thereby confirming its stability.

## Discussion

In conclusion, we developed a new type of liquid scintillator by hybridising colloidal halide perovskite CsPbA_3_ (A: Cl, Br, I) nanocrystals with 2,5-diphenyloxazole (PPO) and demonstrated that the novel liquid scintillator has a very high quantum yield that allows for efficient X-ray detection. Considering their additional advantages, including cost-effective mass production, stability under high-energy X-ray irradiation, and easy processability in combination with various substances, these novel hybrid nanomaterials are suitable as scintillators for a wide range of X-ray technologies that require high-performance detectors and imagers. While the fundamentals of scintillation in these halide perovskite NCs+PPO hybrid nanomaterials require further elucidation, these colloidal hybrid nanocrystals hold substantial promise for advancing the industrial applications of X-ray imaging and producing intriguing scintillation in hybrid nanomaterials.

## Materials and methods

### Chemicals

Caesium carbonate (Cs_2_CO_3_, 99.9%), lead iodide (PbI_2_, 99.9%), lead bromide (PbBr_2_, 99.9%), lead chloride (PbCl_2_, 99.9%), oleic acid (OA, technical grade, 90%), oleylamine (OAm, technical grade 70%), 1-octadecene (ODE, technical grade 90%), n-octane (99%, Germany), and 2,5-diphenyloxazole (PPO, 99%) were purchased from Sigma-Aldrich.

### Preparation of Cs-oleate

The Cs-oleate precursor was synthesised using the conventional hot injection method^26–28^. Cs_2_CO_3_ (0.407 g), OA (1.25 ml) and ODE (15 ml) were dissolved in a 3-necked round-bottom flask by heating under vacuum at 120 °C for 60 min with magnetic stirring. To ensure a complete reaction between Cs_2_CO_3_ and OA, the mixture was heated at 150 °C for 60 min in N_2_.

### Synthesis of CsPbA_3_ (A: Cl, Br, I) nanocrystals and hybrid scintillators

The CsPbA_3_ nanocrystals were prepared using the conventional hot injection method. ODE (25 ml) and 1.89 mmol lead halide (PbI_2_: 0.436 g), lead bromide (PbBr_2_: 0.347 g), or lead chloride (PbCl_2_: 0.263 g) were dissolved in a 3-necked round bottom flask by heating the mixture at 120 °C for 60 min with magnetic stirring under vacuum. Then, the reaction temperature was adjusted from 150–180 °C depending on the lead halide source. Then, the dried OA (2.5 ml) and OAm (2.5 ml) were injected under N_2_. After 30 min, 2 ml of the as-prepared Cs-oleate solution was quickly injected into the reaction mixture solution. As soon as the solution exhibited various colours, corresponding to the perovskite (CsPbA_3_) NCs for each lead halide (PbA_2_), the solution was cooled down in an ice-water bath. The synthesized CsPbA_3_ NC powder was purified by adding hexane and methyl acetate (volume ratio of 1:1) and centrifuged at 8500 rpm. The precipitated CsPbA_3_ NCs were redispersed in octane containing PPO.

### Density functional theory calculations

All ab initio calculations were performed with the Vienna Ab initio Simulation Package (VASP 5.4.4)^39,40^. We used the Perdew–Burke–Ernzerhof (PBE) exchange-correlation functional and the projector augmented-wave (PAW) method^41^. Calculations for geometric optimisation were carried out in a periodically repeated surface (3 × 3) supercell with 1 × 1 *k*-point sampling. A four-layered slab model was employed for CsPbBr_3_ (001), separated by a 15 Å vacuum space in the *z*-direction to avoid interaction between layers. In addition, the two topmost layers were allowed to fully relax, while the other layers were fixed to their optimised bulk positions. A plane-wave cut-off energy of 500 eV was used. Lattice constants and internal atomic positions were fully optimised until the residual forces were <0.04 eV/Å. The schematics of the models are shown in Figs. S5 and S6. To investigate the electronic structures, we employed the Heyd–Scuseria–Ernzerhof (HSE06) hybrid functional calculation^42^ using the GGA-PBE-optimised structures. The solvation free energy (^ΔG^_solv_) calculations were performed using the solvation model based on density (SMD)^43^ at the B3LYP/6-311 + G(2d,p) level of theory with the Gaussian09 package^44^.

### Radioluminescence and X-ray imaging

To evaluate the feasibility of the fabricated scintillation materials in radiation imaging applications, the scintillation characteristics were confirmed under diagnostic X-ray irradiation. The radioluminescence (RL) was measured in the diagnostic energy region of X-rays by varying the tube voltages (10–300 kVp) and currents (5–40 mA) using an X-ray irradiator (X-RAD 320TM, Precision, USA). The amount of radiation absorbed by the scintillation materials was verified by using radiochromic films (Ashland, USA) widely used for radiation dose measurements.

In the therapeutic energy region of X-rays, the RL was also measured using medical linear accelerators for cancer treatment. The RL spectra of the scintillation materials were measured at room temperature under X-ray irradiation. The spectra were recorded using a compact spectrometer that could measure a wavelength range of 200–1000 nm (CCS200, Thorlabs). The scintillation light was transmitted through a Ø200 µm core fibre optically coupled to the spectrometer.

Radiography was performed using a specially designed panel containing the scintillation material (PPO, CsPbA_3_ NCs or PPO + CsPbA_3_ NCs) dissolved in octane. The diameter and thickness of the panel were 4 inches and 1 mm, respectively. The X-ray imaging of the fabricated scintillator was examined for kVp and MVp X-ray irradiation using a medical LINAC (VitalBeam^®^, Varian, USA) equipped with an On-Board Imager^®^ kV imaging system.

### Radiation decay time

The measurement system consisted of two photomultiplier tubes (PMTs) that sensed the scintillation light emitted from the scintillator material. As shown in the figure, PMMA discs with 1 ml sample vials were optically bonded between the two facing PMTs and irradiated with ^60^Co gamma-rays. The signals from the two PMTs were acquired with a 500 MHz FADC to measure the amount and decay time of the scintillation light of all individual events. The event triggering condition was set to be the coincidence of the two channels.

## Supplementary information

Supplementary Information for Hybridisation of perovskite nanocrystals with organic molecules for highly efficient liquid scintillators
