# Supplementary material for: Hybridisation of perovskite nanocrystals with organic molecules for highly efficient liquid scintillators
Source: Light Sci Appl. 2020 Sep 7;9:156. doi: 10.1038/s41377-020-00391-8 (PMC7477552; doi:10.1038/s41377-020-00391-8)
Supplement: Supplementary file 1 — Supplementary Information for Hybridisation of perovskite nanocrystals with organic molecules for highly efficient liquid scintillators [file 41377_2020_391_MOESM1_ESM.pdf]

Supplementary Information for

Hybridisation of perovskite nanocrystals with organic molecules for highly efficient liquid scintillators

Sangeun Cho[1], Sungwoo Kim[2], Jongmin Kim[1], Yongcheol Jo[1], Ilhwan Ryu[3], Seongsu Hong[1], Jae-Joon Lee[3], SeungNam Cha[4], Eun Bi Nam[5], Sang Uck Lee[5], Sam Kyu Noh[1], Hyungsang Kim[1]*, Jungwon Kwak[2]* & Hyunsik Im[1]*

[1] Division of Physics and Semiconductor Science, Dongguk University, Seoul 04620, Korea

[2] Department of Radiation Oncology, Asan Medical Center, Seoul 05505, Korea

[3] Department of Energy and Materials Engineering, Dongguk University, Seoul 04620, Korea

[4] Department of Physics, Sungkyunkwan University, Suwon 2066, Korea

[5] Department of Bionano Technology and Department of Applied Chemistry, Hanyang University, Ansan 15588, Korea

*e-mail: jwkwak0301@gmail.com; hskim@dongguk.edu; hyunsik7@dongguk.edu

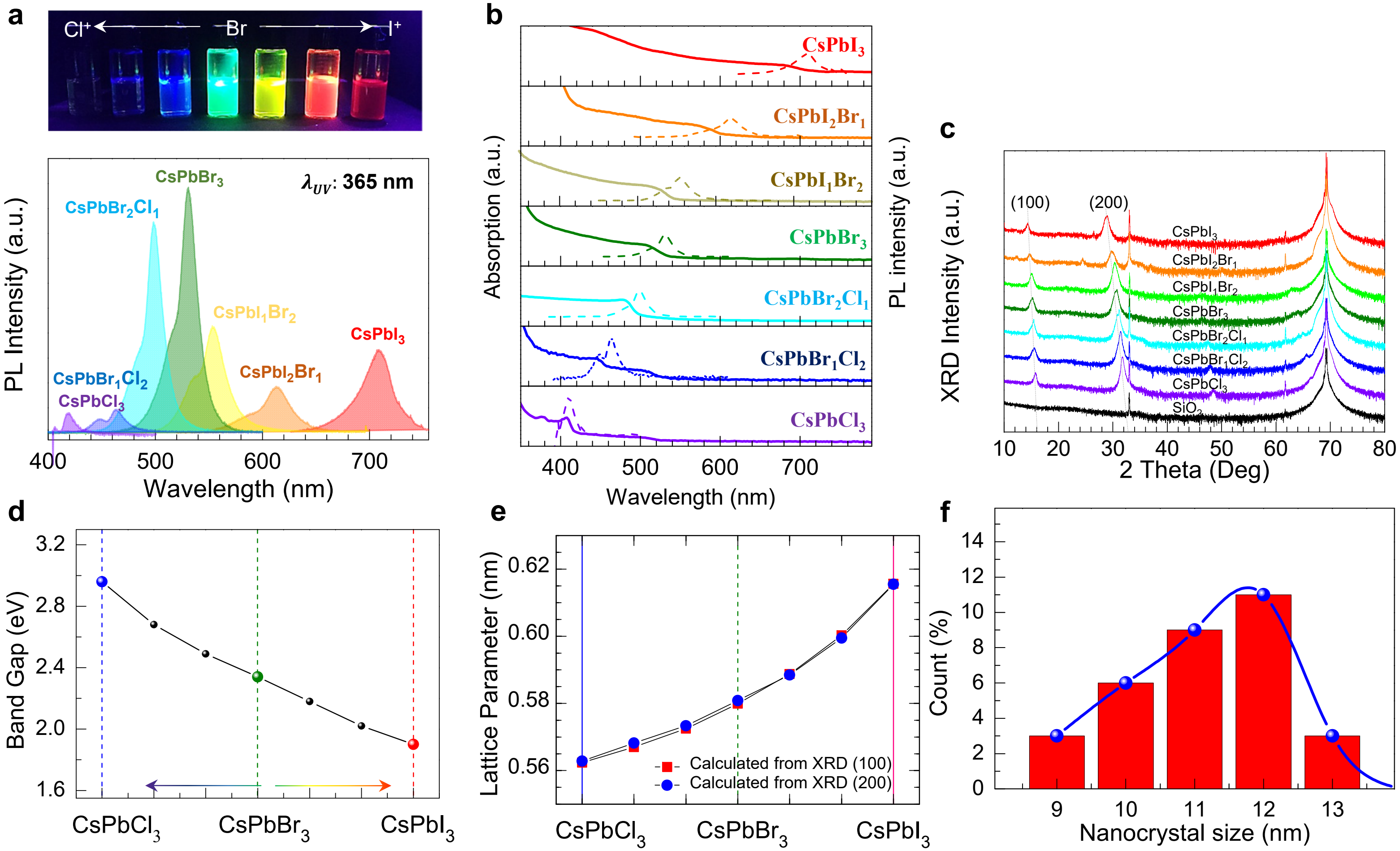

Fig. S1 | Optical properties and Physical characterization of multi-color $CsPbA_3$ NCs scintillators. **a**, Real image and PL spectra of $CsPbA_3$ NCs under UV illumination (λ_{UV}: 365 nm). The asymmetricity of the PL spectra of $CsPbA_3$ NCs is due to an uneven size distribution of $CsPbA_3$ NCs. **b**, UV-Vis spectra. **c**, Band gap of $CsPbA_3$ NCs extracted from the UV-Vis spectra. **d**, X-ray diffraction (XRD) spectra of $CsPbA_3$ NCs. **e**, Calculated lattice parameter of the $CsPbA_3$ NCs using the (100) and (200) peaks of the XRD spectra. **f**, Size distribution of the $CsPbBr_3$ NCs from the TEM image.

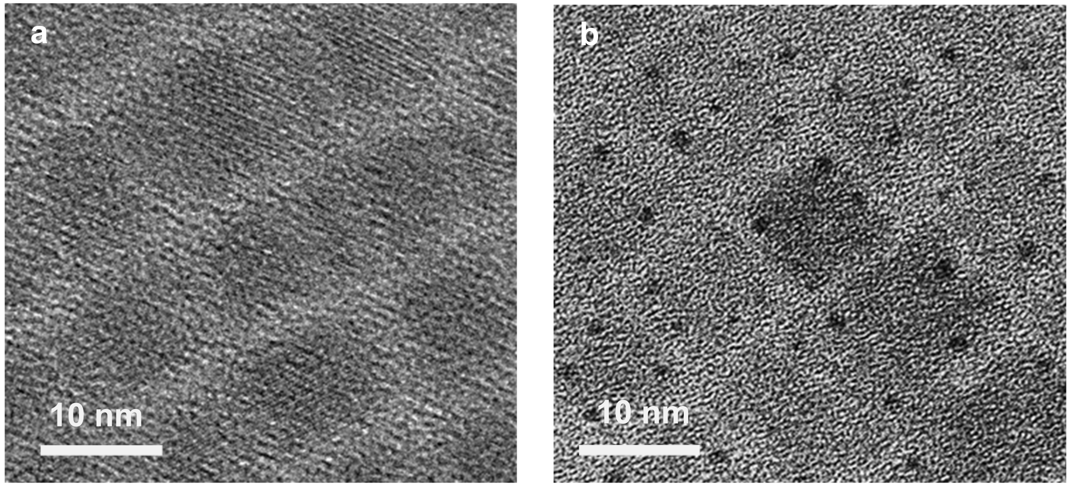

Fig. S2 | TEM images of **a**, $CsPbBr_3$ NCs and **b**, $CsPbBr_3$ NCs+PPO. Dark dots in the TEM images of the $CsPbBr_3$ NCs+PPO sample are associated with organic PPO.

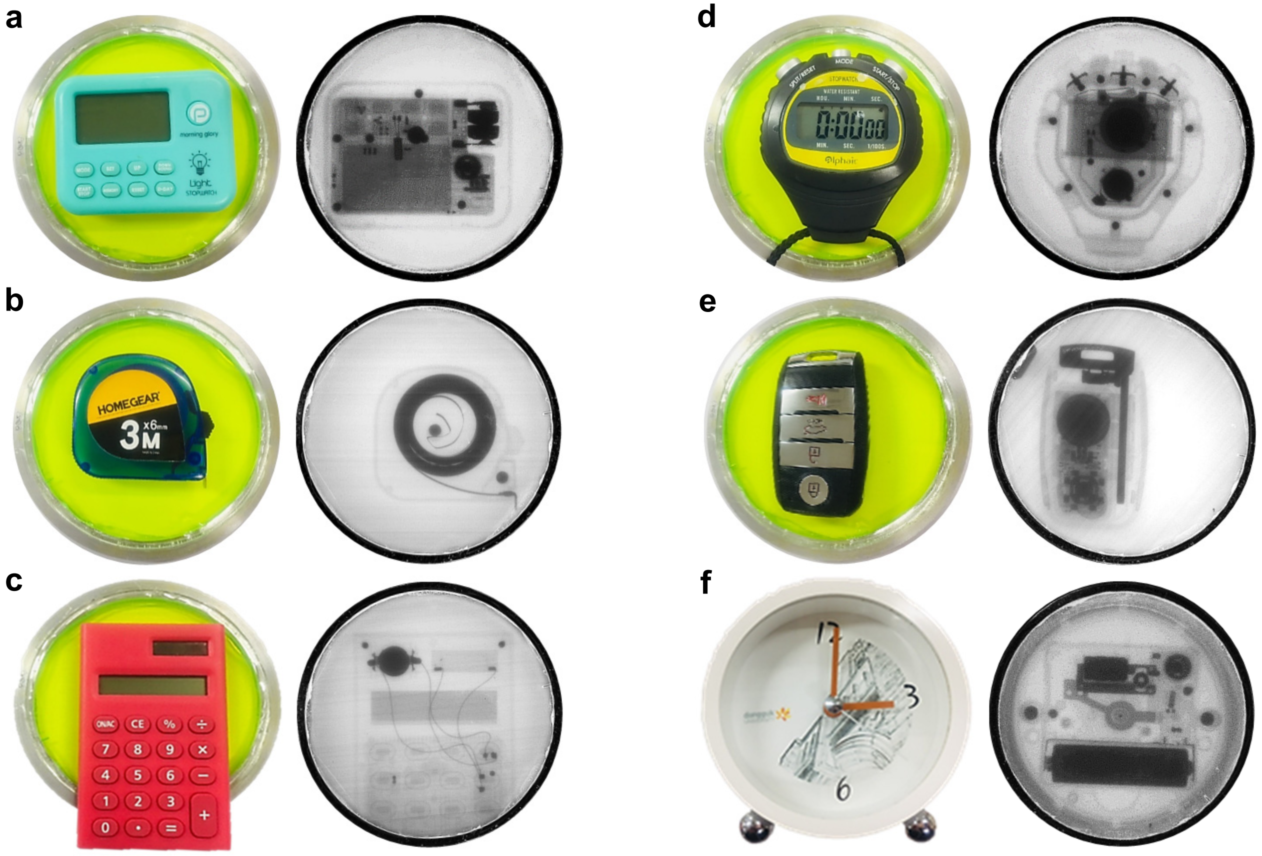

Fig. S3 | X-ray images of various objects. a, Digital timer. **b,** Tape line. **c,** Calculator. **d,** Stop watch. **e,** Car key and **f,** Clock with battery. The X-ray images were recorded using the hybrid $CsPbBr_3$ NCs+PPO scintillator at the same conditions described in Fig. 1 of the main text.

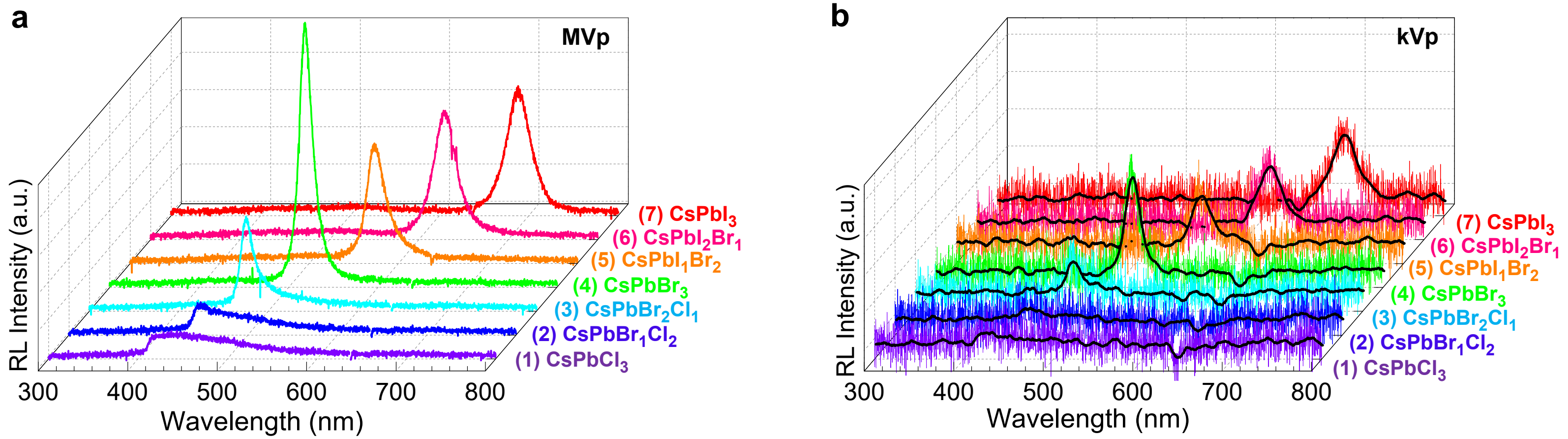

Fig. S4 | RL of hybrid $CsPbA_3$ (A: Cl, Br, I) NCs+PPO scintillators. a, RL spectra in the hard X-ray regime (voltage: 6 MVp) and **b**, RL spectra in the soft X-ray regime (voltage: 140 kVp, 447 $mGy_{air}\ s^{-1}$). $CsPbA_3$ NCs: 25 mg/ml and PPO: 10 mg/ml.

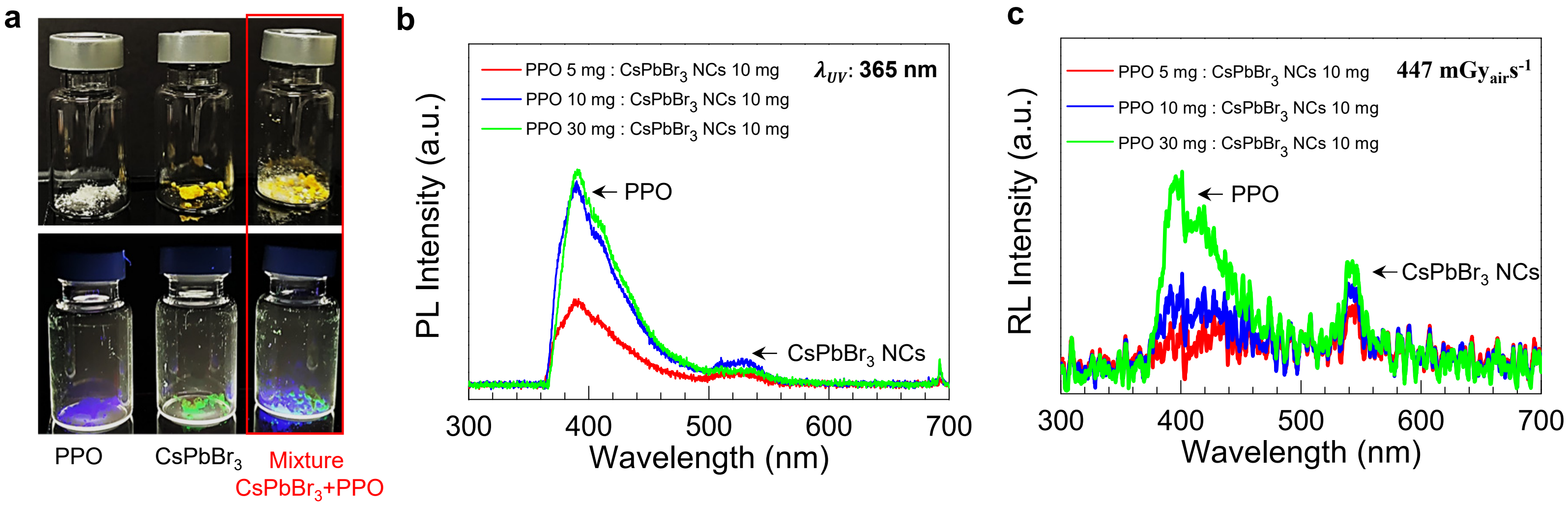

Fig. S5 | PL and RL measurements of a $CsPbBr_3$ NCs+PPO powder mixture without solvent. a, Optical images of the PPO, $CsPbBr_3$ NCs, and $CsPbBr_3$ NCs+PPO mixtures without n-octane under white light (upper column) and UV light (lower column). **b,** PL spectra of a $CsPbBr_3$ NCs+PPO powder mixture sample under UV illumination (λ_{UV}: 365 nm). **c,** RL spectra of a $CsPbBr_3$ NCs+PPO powder mixture sample under X-ray irradiation (447 mGy_{air} s^{-1}). In both the PL and RL spectra, the $CsPbBr_3$ NCs+PPO powder mixture sample clearly shows two peaks that each correspond to PPO and $CsPbBr_3$ NCs, confirming that PPO and $CsPbBr_3$ NCs are not hybridised in ambient air.

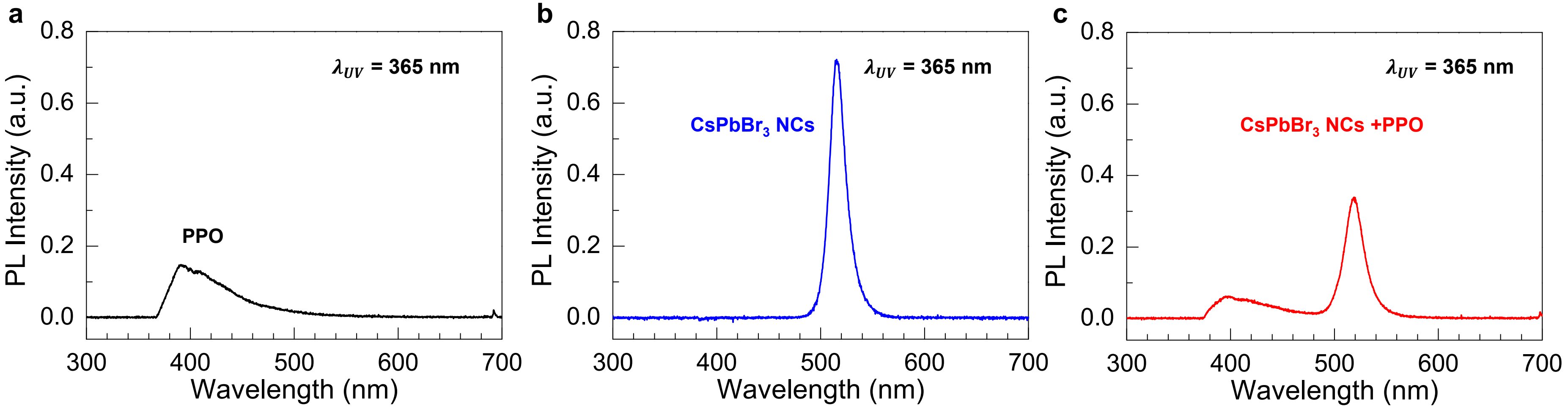

Fig. S6 | PL measurements of PPO, $CsPbBr_3$ NCs, and $CsPbBr_3$ NCs+PPO scintillators in octane. **a**, PPO, **b**, $CsPbBr_3$ NCs and **c**, $CsPbBr_3$ NCs+PPO scintillators under UV irradiation (λ_{UV}= 365 nm).

Ligand	Binding energy (eV)		ΔG_{solv}(Octane) (kcal/mol)
	$CsPbBr_3$ (001):Cs/Br Cs site	$CsPbBr_3$(001):Pb/Br Pb site	
Oleic acid	- 0.16	- 0.30	- 36.05
PPO	- 0.82	- 1.03	- 9.82

Table S1 | Binding energies of PPO and oleic acid (OA) on the Cs and Pb sites of $CsPbBr_3$ (001):Cs/Br and $CsPbBr_3$(001):Pb/Br, respectively, and solvation free energy (ΔG_{solv}) in octane using the solvation model based on density (SMD).

Designing hybrid $CsPbBr_3$+PPO surface structures

For the exploration of X-ray induced charge transfer phenomena on a colloidal hybrid $CsPbBr_3$ NCs+PPO scintillator, we preferentially investigated the PPO binding behaviors on the $CsPbBr_3$ (001) surface, because the $CsPbBr_3$ (001) surface is already well-known to be the most stable among the low index facets in **Fig. S7a**. The $CsPbBr_3$ (001) surface has two different types of surface termination (Cs/Br and Pb/Br terminations), as shown in **Fig. S7b**. Considering the partial charge of eahch atom in PPO in **Fig. S7c**, negatively charged N and O atoms of PPO can electrostatically interact with cationic Cs and Pb sites of $CsPbBr_3$(001) surface. However, bulky phenyl groups hinder the binding of PPO through O atom site. Therefore, we systematically investigated the PPO binding behaviors via N atom considering PPO binding configurations (parallel, tilted and shifted) and the $CsPbBr_3$ (001) surface termination related Cs and Pb binding sites, as shown in **Fig. S8**. The calculated PPO binding energies reveal that the energetically most stable hybrid $CsPbBr_3$ NCs+PPO structure has the N-Pb chemical bonding with shifted binding configuration. Consequently, it is worth mentioning that the N-Pb chemical bond play an important role in finding out the charge transfer mechanism in hybrid $CsPbBr_3$ NCs+PPO scintillator.

In addition, we compared the binding energies of PPO and oleate (OA) on the $CsPbBr_3$ NC surface and how well the desorbed OA can be dissolved in octane solvent. The calculated results reveal that PPO can replace OA bound to the surface of $CsPbBr_3$ NC with higher binding energy, and the desorbed OA can be stabilized in n-octane solvent with negatively large solvation free energy as shown in **Table S1**. Therefore, a colloidal hybrid $CsPbBr_3$ NCs+PPO can be formed well in n-octane solvent by binding of PPO to Pb ion site via N-Pb bonding.

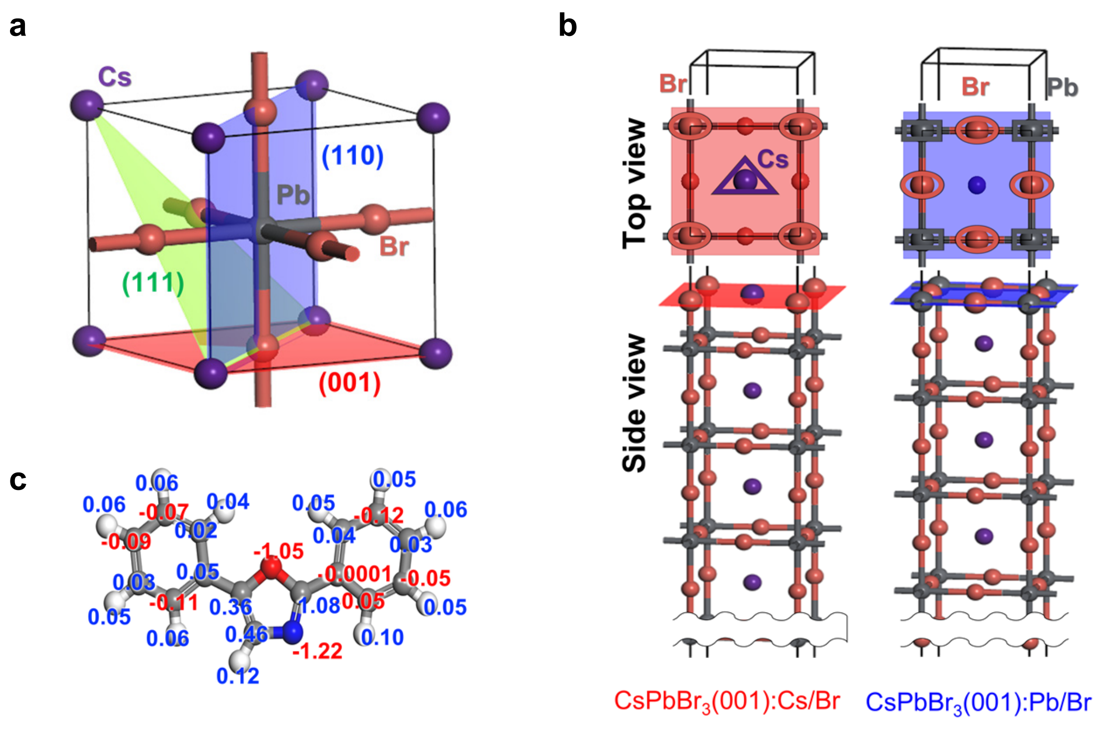

Fig. S7 | $CsPbBr_3$ surface structure and partial charge of each atom in PPO. **a**, Bulk structure of $CsPbBr_3$ with characteristic low index facets. **b**, $CsPbBr_3$ (001) surface structures of different Cs/Br and Pb/Br surface types. **c**, Partial charge of each atom in 2,5-diphenyloxazole (PPO).

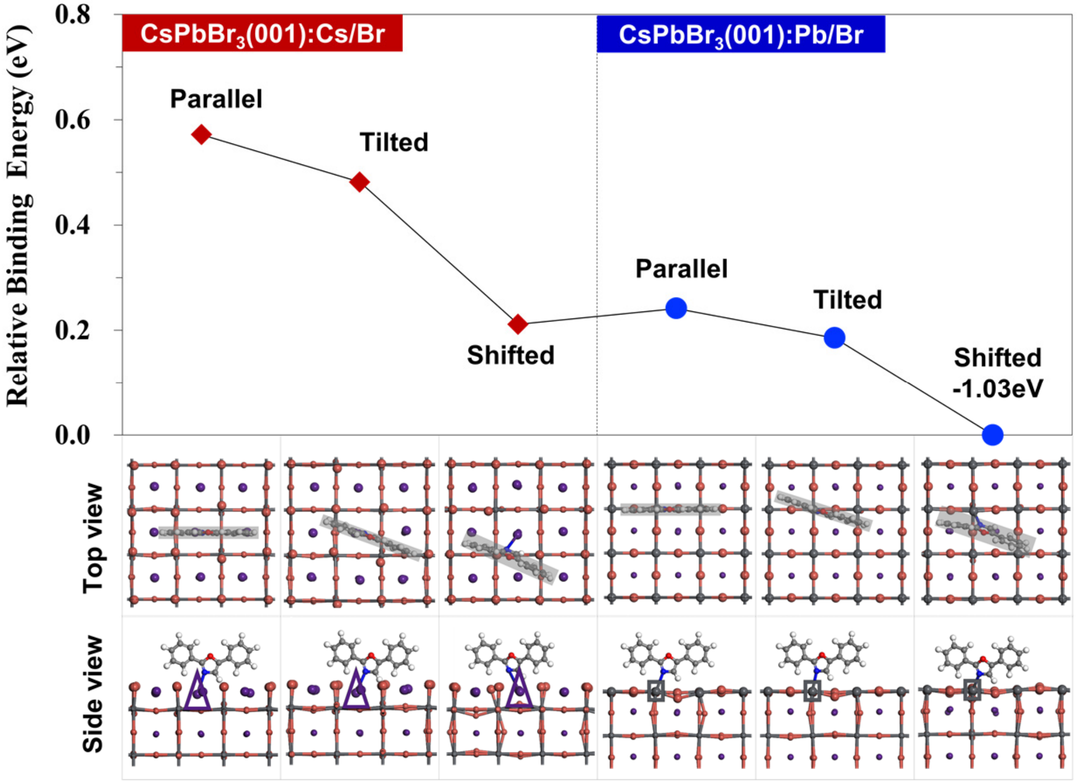

Fig. S8 | Relative PPO binding energies depending on binding configurations and $CsPbBr_3$ (001) surface types. Relative PPO binding energies on $CsPbBr_3$(001):Cs/Br and $CsPbBr_3$(001):Pb/Br surfaces with parallel, tilted and shifted binding configurations. The PPO binding energy (E_b) is calculated by $E_b = E_{CsPbBr_3/PPO} - (E_{CsPbBr_3} + E_{PPO})$. E_{CsPbBr_3} and E_{PPO} are the total energy of the E_{CsPbBr_3} (001) surface structure and the isolated E_{PPO} structure, respectively.

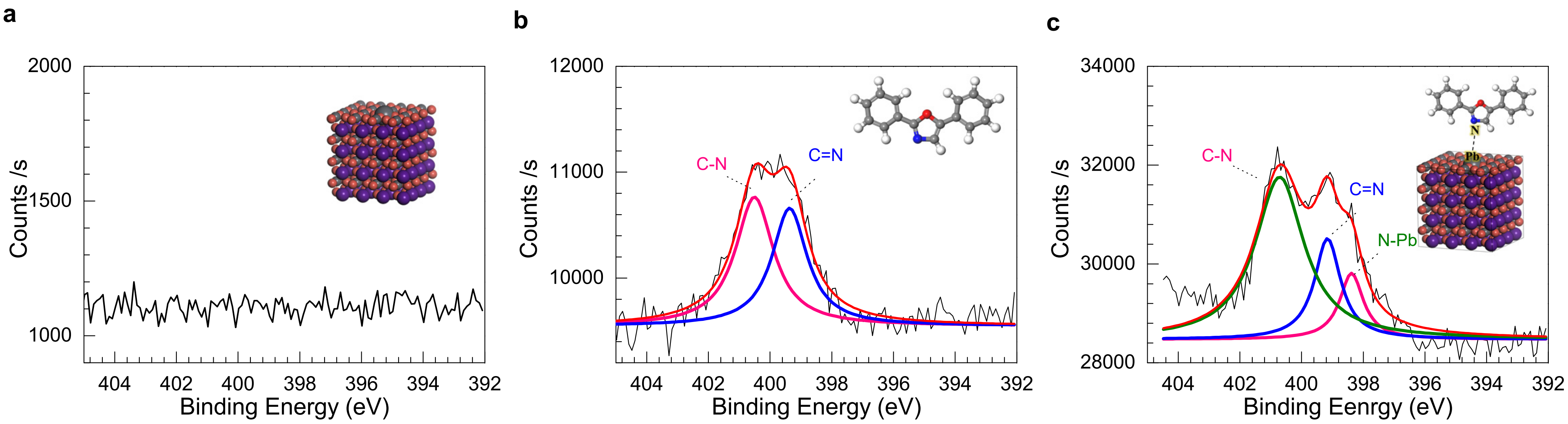

Fig. S9 | XPS measurements (N1s spectra). a, $CsPbBr_3$ NCs. **b**, PPO, **c**, $CsPbBr_3$ NCs + PPO. The peaks observed at 400.7 eV, 399.1 eV, and 398.2 eV correspond to C-N, C=N, and N-Pb bonding, respectively [Z. Wei, et. al., Front. Chem., 7, 645 (2019)]. As anticipated, any N1s signal is not detected in $CsPbBr_3$ NCs. In the presence of PPO, a clear signature for N-Pb bonding is detected at 398.2 eV in the N1s spectrum of the $CsPbBr_3$ NCs+PPO sample.

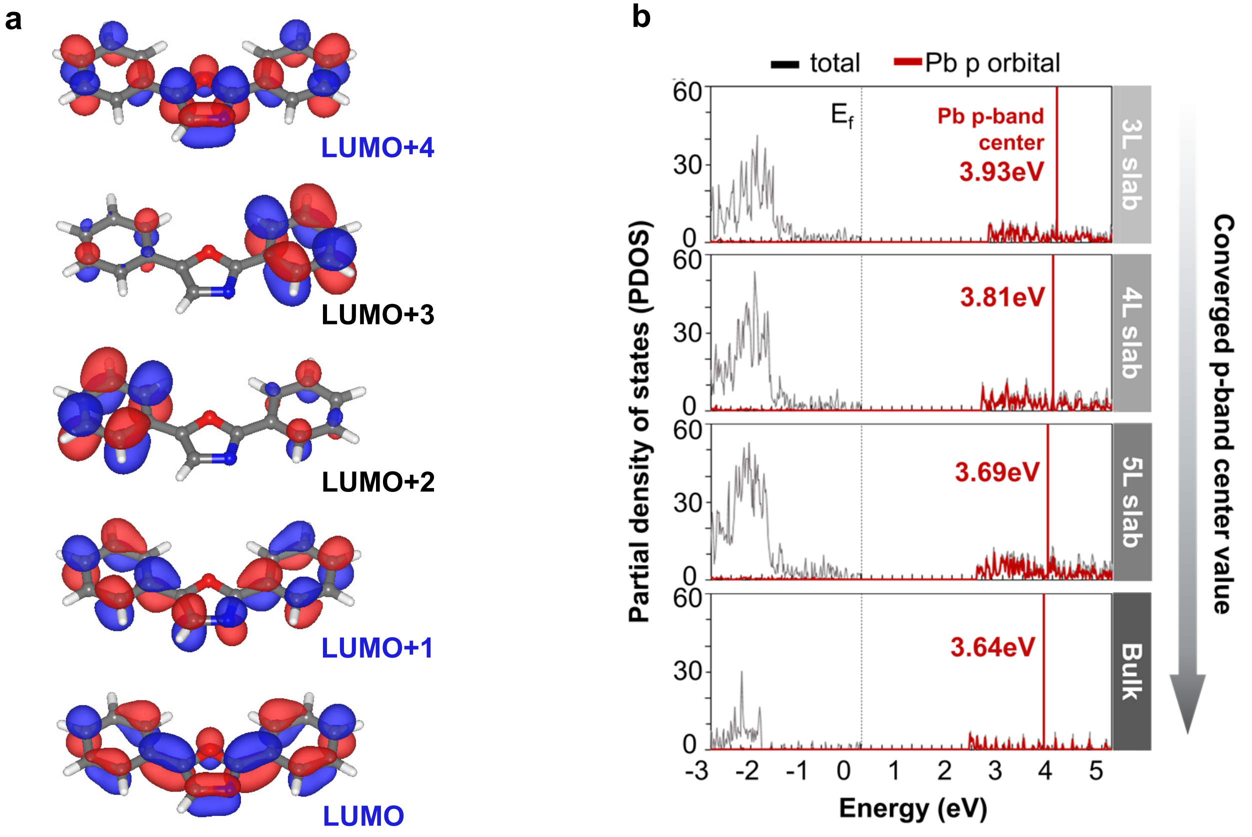

Fig. S10 | Frontier molecular orbitals of PPO and partial density of state (PDOS) of the Pb *p*-orbital in the $CsPbBr_3$ (001) surface structure. a, The frontier molecular orbital distributions (0.03 isosurface value) of 2,5-diphenyloxazole (PPO) calculated at M062X/6-31G* level of theory. **b**, Partial density of states (PDOS) of the Pb *p*-orbital in the $CsPbBr_3$ (001) surface slab models with different slab thicknesses , from 3-layers (3L) to 5 layers (5L) and bulk. The black and red lines represent total and Pb 6*p* states, respectively. The Fermi level is indicated by a black dashed line.

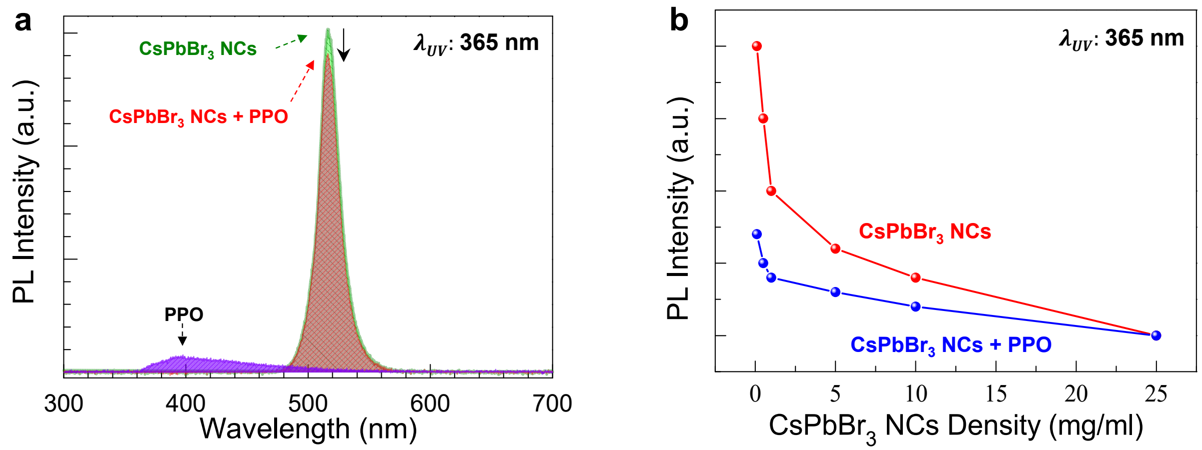

Fig. S11 | Comparison of UV-induced luminescence for PPO, $CsPbBr_3$ NCs and hybrid $CsPbBr_3$ NCs +PPO scintillators. a, PL spectra of the PPO, $CsPbBr_3$ NCs and hybrid $CsPbBr_3$ NCs +PPO scintillators under UV illumination (λ_{UV}: 365 nm). PPO: 10 mg/ml, $CsPbBr_3$ NCs : 25 mg/ml. **b**, Intensity of PL spectra for the $CsPbBr_3$ NCs and hybrid $CsPbBr_3$ NCs +PPO scintillators under UV illumination as a function of NCs density. PPO: 10 mg/ml. PPO does not contribute to the enhancement of the photoluminescence of the hybrid $CsPbBr_3$ NCs +PPO scintillator under low-energy UV illumination, regardless of NCs density.

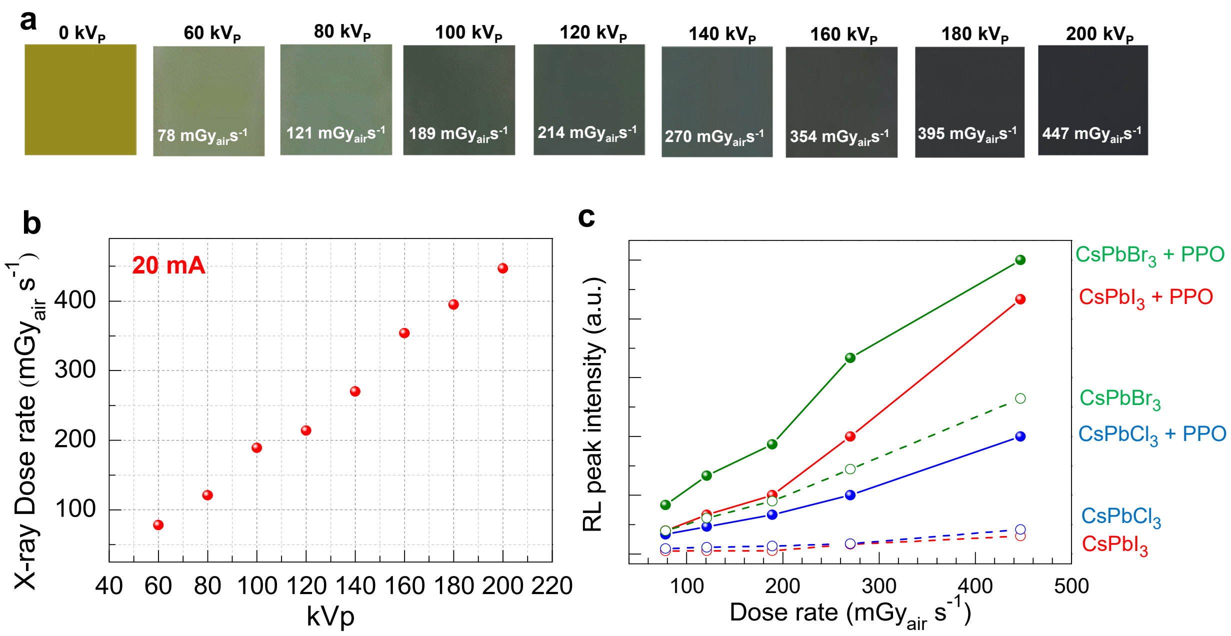

Fig. S12 | RL measurements of the colloidal hybrid $CsPbA_3$ NCs+PPO and $CsPbA_3$ (A: Cl, Br, I) NCs as a function of dose rate. a, Measurements of X-ray dose rate as a function of acceleration voltage (kVp) using Gafchromic EBT3 films. **b**, Extracted dose rate as a function of acceleration voltage (kVp) at a current density of 20 mA. **c,** Intensity of RL for hybrid $CsPbA_3$ NCs+PPO and $CsPbA_3$ NCs as a function of dose rate. The measured RL peak intensity of the samples increases linearly with increasing dose rate.

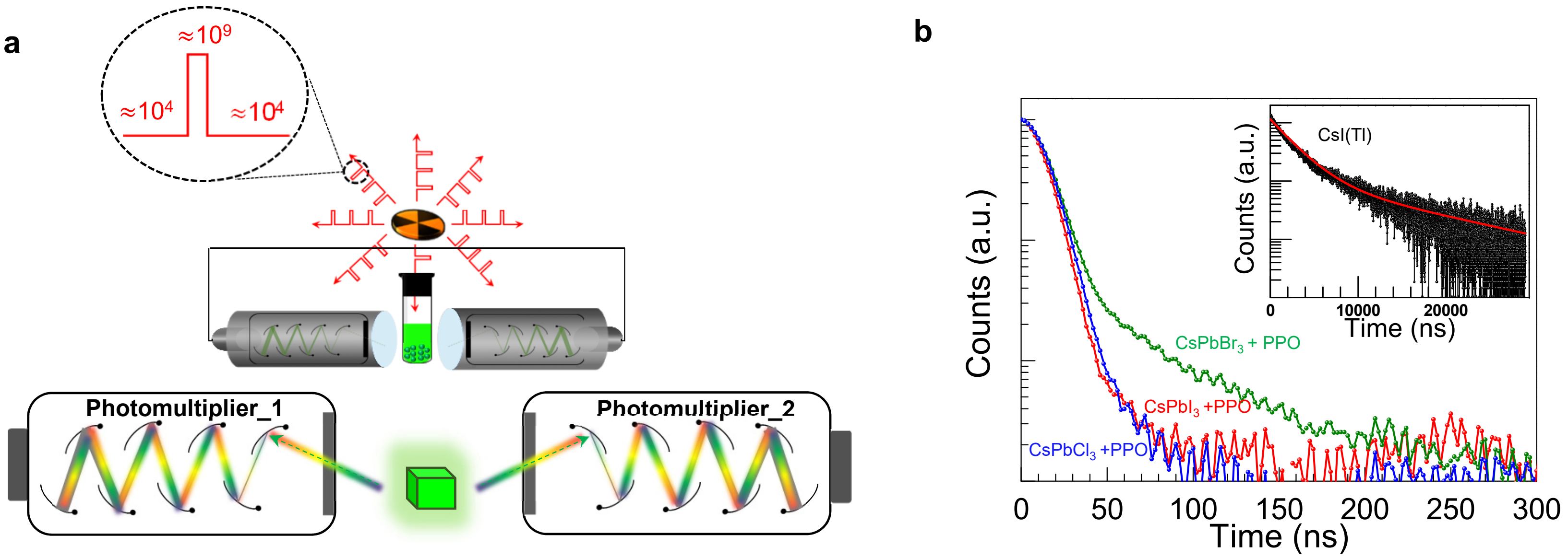

Fig. S13 | Measurements of X-ray scintillation decay time. a, Experimental setup for measuring X-ray scintillation decay time. The measurement system consists of two photomultiplier tube (PMT) channels, a coincidence trigger logic and Flash Analog Digital Converter (FADC). As shown in the figure, the 1 ml sample vials inserted into the transparent PMMA disc were optically bonded between two facing PMTs and were irradiated by ^{60}Co gamma-rays. The signals from the PMTs were taken via two 500MHz FADC channels under coincidence condition to acquire the amount and the decay time of the scintillation lights of every single event. The event trigger threshold was set to be sufficiently bigger than the amplitude of a single photon signal and PMT dark noise. Single channel signals rather than the summed signals were employed for the decay time measurement. **b**, The spectra collected from the scintillation counting for the colloidal hybrid $CsPbA_3$ (A: Cl, Br, I) NCs+PPO samples for a gamma-ray source (^{60}Co). Bulk CsI:Tl is included for comparison. The spectra are normalized.

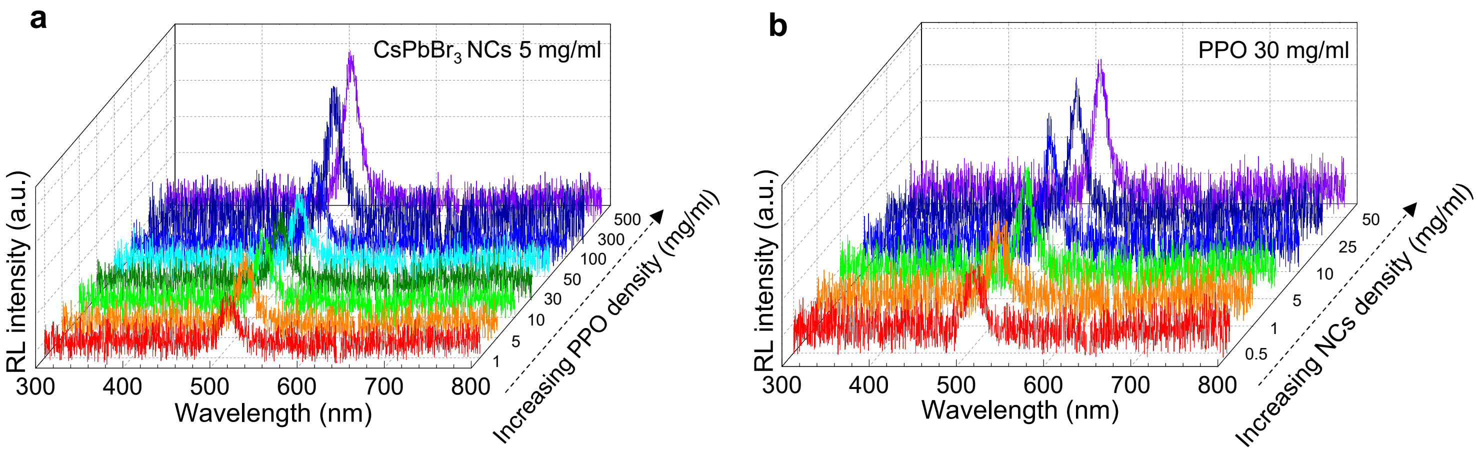

Fig. S14 | RL of hybrid $CsPbBr_3$ NCs+PPO scintillators in the soft X-ray regime (Voltage: 70 kVp, 37.4 $mGy_{air}\ s^{-1}$). a, RL spectra of the hybrid $CsPbBr_3$ NCs+PPO scintillators with increasing PPO density at a fixed NC density (5 mg/ml). **b**, RL spectra of the hybrid $CsPbBr_3$ NCs+PPO scintillators with increasing NCs density at a fixed PPO density (30 mg/ml).

a

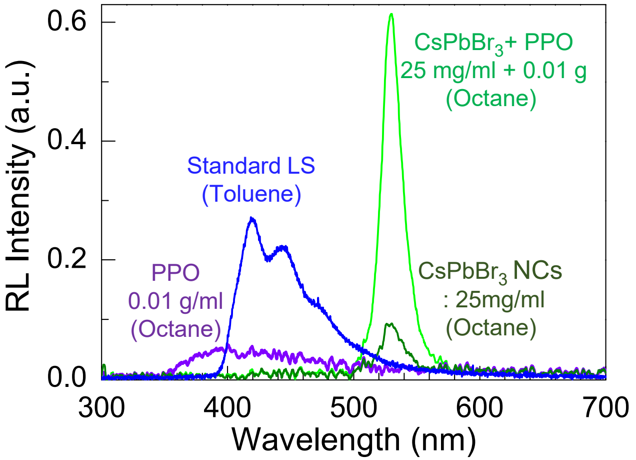

b

Scintillator	Light yield (Photons/keV)	
CsI(Tl) : Commercial Solid	54.0	473 %
NaI(Tl) : Commercial Solid	38.0	330 %
Standard LS: PPO + POPOP + toluene	11.4	100 %
Hybrid LS : NCs(25mg/ml)+PPO (0.5g)	23.3	204 %
Hybrid LS : NCs(25mg/ml)+PPO (0.01g)	9.5	83 %
PPO (0.01g)	4.8	42 %
$CsPbBr_3$ NCs (25 mg/ml)	2.3	25 %

c

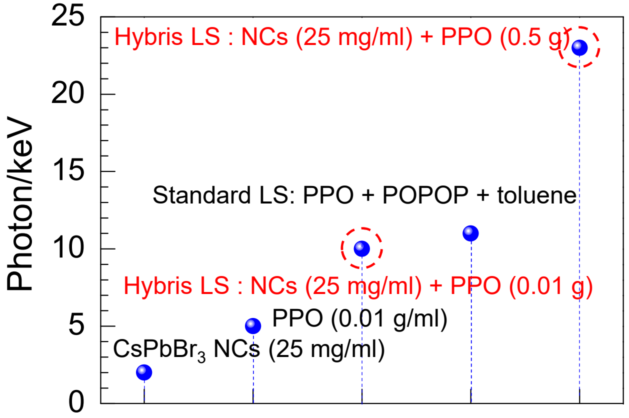

Fig. S15 | X-ray scintillator efficiency a, RL spectra of standard liquid scintillator (PPO+POPOP+toluene), and our scintillators (PPO, $CsPbBr_3$ NCs, and $CsPbBr_3$ NCs+PPO in octane.) measured at 6 MeV. **b**, Comparison of the X-ray light yield of various solid and liquid scintillators. LS: liquid scintillator. The relative efficiency values of the solid scintillators (CsI and NaI) were obtained from Ref. (Tsipenyuk, Y. M. Physical methods, instruments and measurements, Volume II, p. 71). Note that scintillation efficiency of $CsPbBr_3$+PPO was enhanced with increasing PPO density. **c**, Light yield (photons/keV) obtained from various liquid scintillators. The light yield of the standard PPO+POPOP (toluene) is known and the light yield values of other liquid scintillators were evaluated with respect to that of the standard PPO+POPOP (toluene) scintillator.

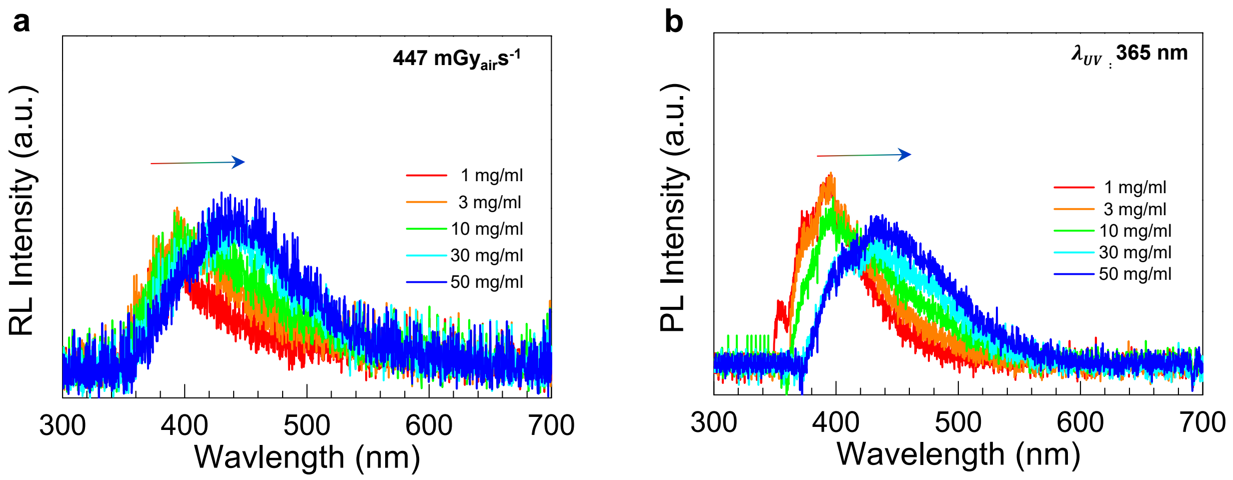

Fig. S16 | Self-absorption of pure PPO. a, RL spectra of pure PPO in octane with increasing PPO density. **b**, PL spectra of pure PPO in octane with increasing PPO density. As the PPO density increases the RL and PL peaks are red-shifted and their peak intensities decrease due to self-absorption.

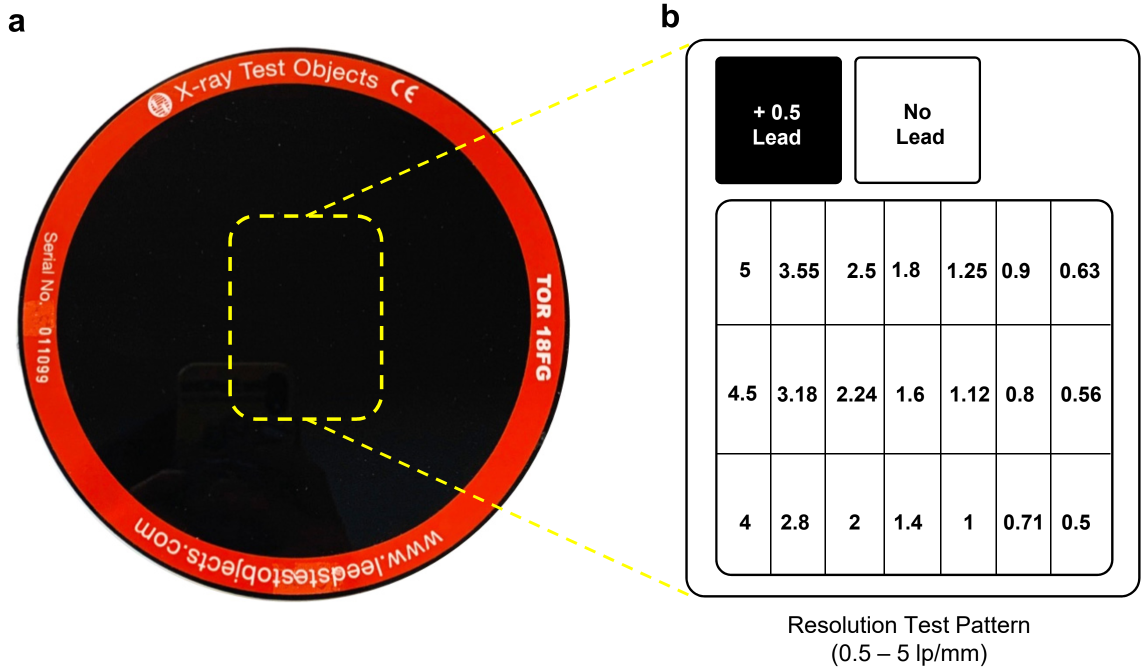

Fig. S17 | Leeds Test objects. a, Real image of the Test Phantom used for X-ray image evaluation. **b**, Schematic representation of the hidden pattern in the Leeds Test Objects. Numbers (0.5–5) represent line pairs per millimetre (lp/mm).

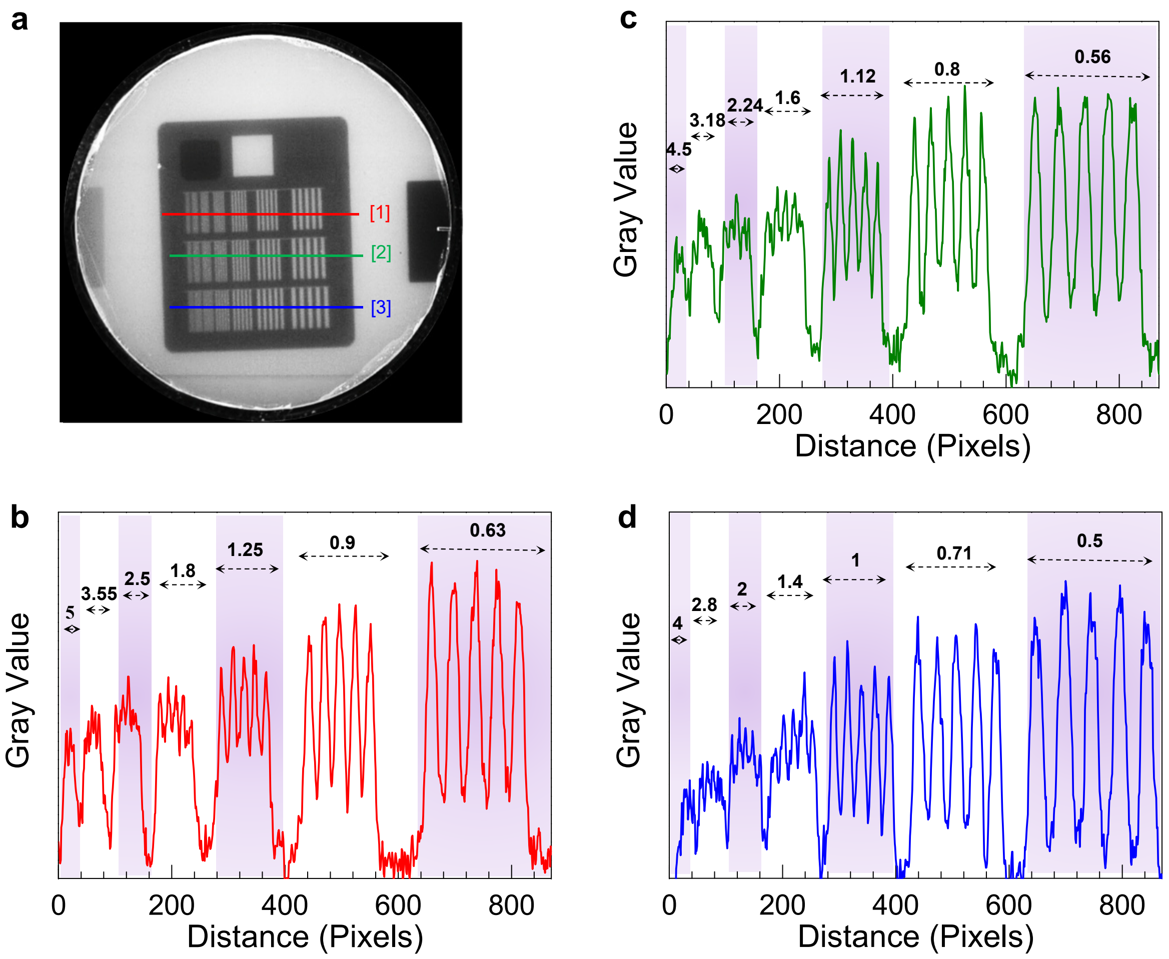

Fig. S18 | X-ray image of the test objects using the same hybrid liquid scintillator panel detector after a year. a, X-ray image of the Leeds test objects recorded a year later. **b-d**, X-ray line pair profiles along the color lines in **a**.

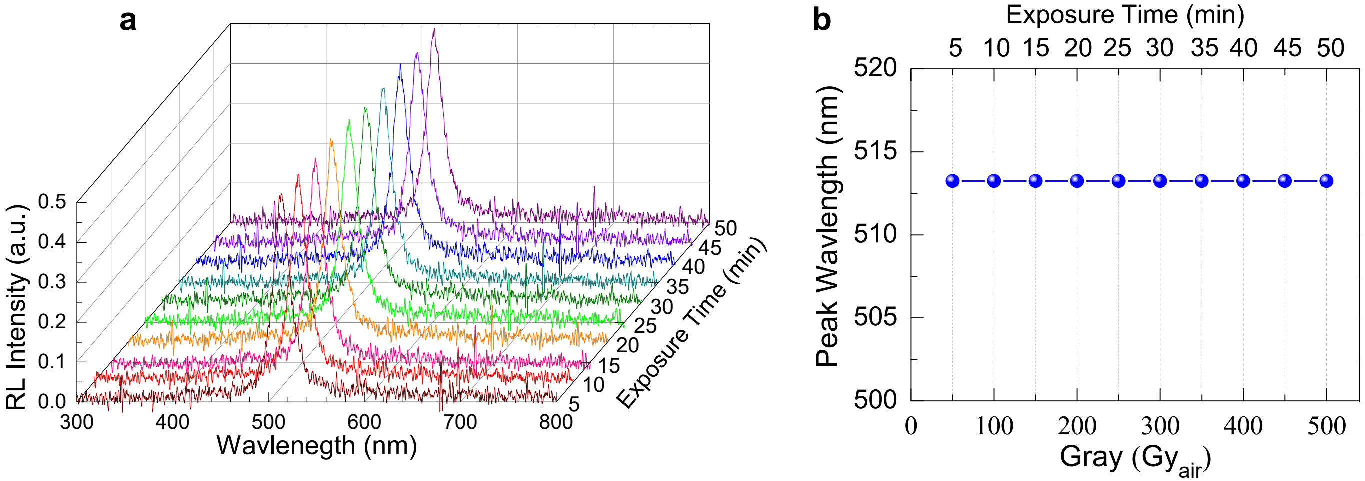

Fig. S19 | a, Radioluminescence spectra of a hybrid $CsPbBr_3$ NCs+PPO scintillator for a prolonged period at very high energy X-ray (10 MeV). **b**, Peak position of the measured RL spectra as a function of dose rate. The hybrid scintillator was exposed to a very high energy X-ray (10 MeV) continuously for 50 min and its RL was measured every 5 min (5 min × 10 times). The RL intensity and peak position remain almost similar confirming its excellent stability even at high dose rate (10 MeV for 5 min: 50 Gy_{air}).
